# Human Lung Mast Cells: Therapeutic Implications in Asthma

**DOI:** 10.3390/ijms232214466

**Published:** 2022-11-21

**Authors:** Remo Poto, Gjada Criscuolo, Gianni Marone, Chris E. Brightling, Gilda Varricchi

**Affiliations:** 1 Department of Translational Medical Sciences, University of Naples Federico II, 80131 Naples, Italy; 2 Center for Basic and Clinical Immunology Research (CISI), University of Naples Federico II, 80131 Naples, Italy; 3 World Allergy Organization (WAO), Center of Excellence (CoE), 80131 Naples, Italy; 4 Institute of Experimental Endocrinology and Oncology “G. Salvatore”, National Research Council (CNR), 80131 Naples, Italy; 5 Department of Respiratory Sciences, Leicester NIHR BRC, Institute for Lung Health, University of Leicester, Leicester LE1 7RH, UK

**Keywords:** airway remodeling, asthma, basophil, biological therapies, eosinophil, macrophage, mast cell

## Abstract

Mast cells are strategically located in different compartments of the lung in asthmatic patients. These cells are widely recognized as central effectors and immunomodulators in different asthma phenotypes. Mast cell mediators activate a wide spectrum of cells of the innate and adaptive immune system during airway inflammation. Moreover, these cells modulate the activities of several structural cells (i.e., fibroblasts, airway smooth muscle cells, bronchial epithelial and goblet cells, and endothelial cells) in the human lung. These findings indicate that lung mast cells and their mediators significantly contribute to the immune induction of airway remodeling in severe asthma. Therapies targeting mast cell mediators and/or their receptors, including monoclonal antibodies targeting IgE, IL-4/IL-13, IL-5/IL-5Rα, IL-4Rα, TSLP, and IL-33, have been found safe and effective in the treatment of different phenotypes of asthma. Moreover, agonists of inhibitory receptors expressed by human mast cells (Siglec-8, Siglec-6) are under investigation for asthma treatment. Increasing evidence suggests that different approaches to depleting mast cells show promising results in severe asthma treatment. Novel treatments targeting mast cells can presumably change the course of the disease and induce drug-free remission in bronchial asthma. Here, we provide an overview of current and promising treatments for asthma that directly or indirectly target lung mast cells.

## 1. Introduction

Mast cells, first identified in humans by Paul Ehrlich in 1878 [1], play a role in allergic [2,3] and autoimmune disorders [4], microbial infections [5], cardiovascular diseases [6,7], immunodeficiencies [8], and cancer [9,10,11]. Mast cells are derived from CD34^+^ haemopoietic progenitors that migrate from the bone marrow to the blood and mature in almost all tissues [12]. These cells release a plethora of mediators and display several surface receptors [13,14]. Mast cells uniquely express the cell surface receptor of stem cell factor (SCF) [15], also known as KIT or CD117. SCF plays a critical role in the differentiation, proliferation, and modulation of human and rodent mast cells [16].

In the 1990s, human mast cells that contain only tryptase were termed MC_T_, whereas those that express both tryptase and chymase were classified as MC_TC_ [17,18]. There are also definitions of mast cells being inflammatory, pro-, or anti-tumorigenic [19,20,21]. The transcriptional profiles of mast cells clearly demonstrate the heterogeneity of mast cells and their different gene expression [14,22,23,24]. Moreover, human mast cells analyzed ex vivo or differentiated in vitro showed significant differences [24]. Human and mouse mast cells have distinct proteomes and unique gene expressions compared to other immune cells [22,25]. Single-cell transcriptomics of human lungs provide evidence of mast cells [26,27]. Different triggers (e.g., IgE-mediated or IL-33) can induce distinct genomic and transcriptional changes in human mast cells [28]. Individual mast cells are exposed to their local environment (e.g., cytokines, different pH, growth factors, etc.) and, over time, are tuned by many different activating and inhibitory signals. Mast cells in different organs differ in their receptor and mediator expression, but there is also considerable heterogeneity among human lung mast cells [29]. It is possible to speculate that individual mast cells could all be unique to some extent.

In this review, we provide an overview of current and promising treatments for asthma that directly or indirectly target lung mast cells.

## 2. Activating and Inhibitory Receptors on Human Mast Cells

**Figure 1:**
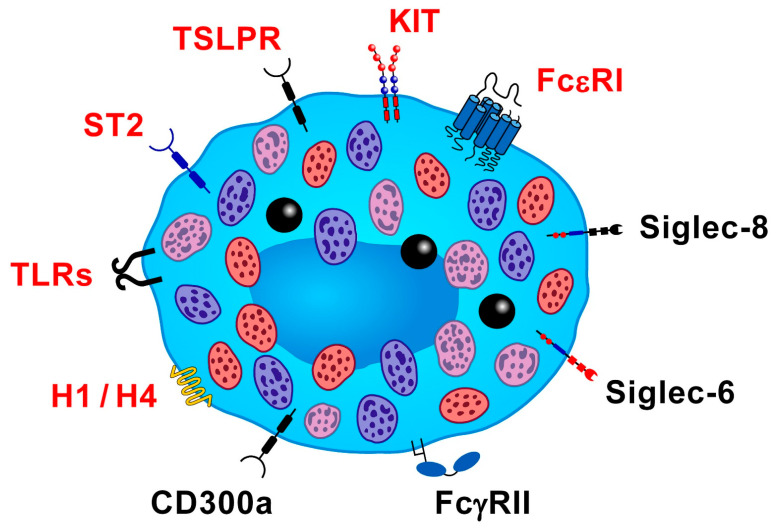
Schematic representation of activating (in red) and inhibitory (in black) receptors on human lung mast cells. A complete (αβγ2), high-affinity receptor for IgE (FcεRI) is expressed by human mast cells and basophils [31]. Antigens, superantigens, and anti-IgE lead to mast cell activation and mediator release through the aggregation of FcεRI bound to IgE [31,32,33]. Mast cells play a role in allergic disorders by releasing preformed (e.g., histamine, tryptase) and de novo synthesized mediators (e.g., cysteinyl leukotriene C_4_ (LTC_4_), prostaglandin D_2_ (PGD_2_)) and several cytokines and chemokines [3,19]. SCF, which activates the KIT receptor (CD117), highly expressed by mast cells throughout their development, is the most important factor regulating these cells [34,35]. SCF is released by a plethora of cells, including fibroblasts, endothelial cells, and mast cells themselves [36,37]. Thymic stromal lymphopoietin (TSLP) is expressed by airway epithelial cells [38], keratinocytes [39], immune and structural cells [40] and acts as an alarmin. TSLP activates the heterodimeric receptor comprising IL-7Rα and TSLP receptor (TSLPR) on mast cells and other immune cells [41,42,43]. TSLPR mRNA, but not IL-7Rα mRNA, is expressed by human mast cells [42,44]. TSLP, under certain experimental conditions [42], can induce the release of chemokines and cytokines from mast cells [45]. IL-33 is released by damaged epithelial and endothelial cells [46,47] and activates mast cells [48,49]. IL-33 engages the heterodimeric receptor, ST2-IL-1RAcP, on human mast cells [50,51,52] and induces cytokine [50,53,54,55,56] and chemokine release [57]. IL-33 and superantigenic activation cause the release of angiogenic and lymphangiogenic factors from human lung mast cells (HLMCs) [58]. Different mast cell subsets display distinct toll-like receptors (TLRs) [59]. Activation of TLR2, -3, -4, -6, -7, and -9 induce cytokine release from human mast cells [60,61,62]. FcεRI cross-linking amplifies TLR-induced cytokine released from human mast cells [63]. Histamine is preformed in cytoplasmic granules of human mast cells (≅3 pg/cell) and basophils (≅1 pg/cell) [64,65]. Human mast cells express the histamine H1 and H4 receptors [66]. Histamine induces exocytosis and IL-6 production from human lung macrophages by activating H1 receptors [67]. High concentrations of certain H1-antihistamines can inhibit mediator release from human FcεRI^+^ cells [68,69]. H4R is expressed by human mast cells, and its activation can modulate the function of these cells [66]. Mast cells display several inhibitory receptors [70,71], such as CD300a and CD200R [72,73]. Coaggregation of CD300a and FcεRI with a bispecific antibody inhibits IgE-mediated tryptase and IL-4 release from human mast cells [74] and IgE-mediated anaphylaxis in preclinical asthma models [75]. Siglecs are inhibitory receptors expressed on immune cells [76]. Siglec-8 is expressed on murine and human mast cells [77,78] and on mast cell lines [76,77,79]. Siglec-8 monoclonal antibody (mAb) diminishes FcεRI-mediated histamine and PGD_2_ release from mast cells [80]. Siglec-8 is also present on eosinophils and basophils [81,82]. Siglec-6 is selectively expressed by mast cells [76], and a mAb cross-linking this receptor (AK006) potently inhibits IgE-mediated human mast cell activation. Siglec-6 interacts with KIT/CD117 and inhibits SCF-mediated mast cell activation (Korver, Schanin, 10th EMBRN meeting, July 11–12, 2022). Human mast cells express the activating FcγRIIA and FcγRI induced by IFN-γ [83]. A human IgG-IgE Fc fusion protein co-crosslinks FcεRI and FcγRII receptors and inhibits histamine release from human basophils and HLMCs [84,85,86]. A dual-targeting tandem IgE-IgG Fc domain inhibits mast cell degranulation [87].

Human mast cells display a wide spectrum of cell surface receptors that can be activated by several immunologic and non-immunologic stimuli that modulate their development and effector functions [11,30]. Figure 1 schematically illustrates the main activating and inhibitory receptors on human lung mast cells relevant to bronchial asthma.

Activation of human lung mast cells releases a vast arsenal of preformed and newly synthesized lipid mediators, cytokines, chemokines, angiogenic, and lymphangiogenic factors [88] (Table 1).

## 3. Role of Mast Cells in Asthma

FcεRI cross-linking by allergens, anti-IgE, or super allergens results in the release of histamine, cytokines/chemokines, enzymes such as tryptase and chymase, and the generation of eicosanoids (i.e., LTC_4_ and PGD_2_) from human mast cells [32,134]. Mast cell-derived mediators are responsible for bronchoconstriction, airway inflammation, and remodeling in different asthma endotypes [2]. The density of mast cells within airway smooth muscle (ASM) bundles is increased in asthmatic patients compared to controls [90]. There is an inverse correlation between the number of mast cells in the ASM and airway hyperresponsiveness (AHR) in asthmatics [90]. HLMCs adhere avidly to ASM cells [135], which favor mast cell survival and activation [136]. Elevated circulating mast cell progenitors are correlated with reduced lung function in allergic asthma [137]. The rapid IgE-dependent release of histamine and eicosanoids (e.g., LTC_4_ and PGD_2_) from isolated HLMCs [138] correlates with these mediators in bronchoalveolar lavage fluids following bronchial allergen challenge [139,140,141]. Histamine can promote mucus secretion and bronchoconstriction. Asthma is accompanied by airway remodeling [142] and angiogenesis [143,144], and lung mast cells may contribute to this by the release of several cytokines, chemokines [88], and VEGFs [58,126,145,146]. Submucosal mast cells, which are abundant in healthy controls, are shifted from the submucosal compartment to the epithelium in asthma [147]. IL-33-activated mast cells increase the expression of epithelial *IL33*, which in turn upregulates the production of type-2 cytokines (i.e., IL-5, IL-13, IL-4) in mast cells. These findings demonstrate a shift in the location of mast cells to the epithelium in asthma and identify intraepithelial mast cells as critical modulators of inflammation in asthma. Psychological stress is thought to induce mast cell activation via the stimulation of peripheral nerves and the release of substance P and corticotropin-release hormone (CRH) [148]. Human mast cells express CRH receptors and their activation induces the selective release of VEGF-A without degranulation [149]. These findings provide a hypothetical link between stress, mast cell activation, and asthma exacerbations [150]. The role of HLMCs in inducing the symptoms of human airway inflammation is also supported by the efficacy of drugs, which block either their function or target mediators primarily released by these cells.

**Figure 2:**
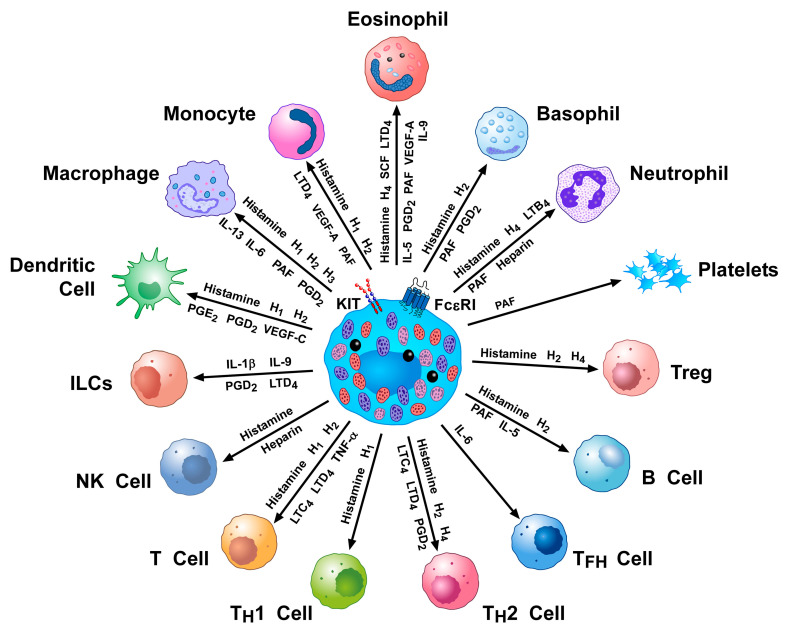
Schematic representation of the multiple interactions between human lung mast cells and several cells of the innate and adaptive immune system through the release of mediators. Mast cells can interact with monocytes (histamine, LTD_4_, VEGF-A, and PAF), macrophages (histamine, IL-13, IL-6, PAF, and PGD_2_), dendritic cells (DCs) (histamine, PGE_2_, PGD_2_, VEGF-C, and IL-13), ILCs (IL-1β, IL-9, PGD_2_, and LTD_4_), NK cells (histamine and heparin), T cells (histamine, LTC_4_, LTD_4_, and TNF-α), T_H_1 (histamine) and T_H_2 (histamine, LTC_4_, LTD_4_, and PGD_2_) cells, T_FH_ cells (IL-6), B cells (histamine, PAF, and IL-5), Treg cells (histamine), eosinophils (histamine, IL-5, IL-9, SCF, LTD_4_, PAF, PGD_2_, and VEGF-A), neutrophils (histamine, LTB_4_, PAF, and heparin), basophils (histamine, PAF, and PGD_2_), and platelets (PAF). Modified with permission from Varricchi 2019 [151].

**Figure 3:**
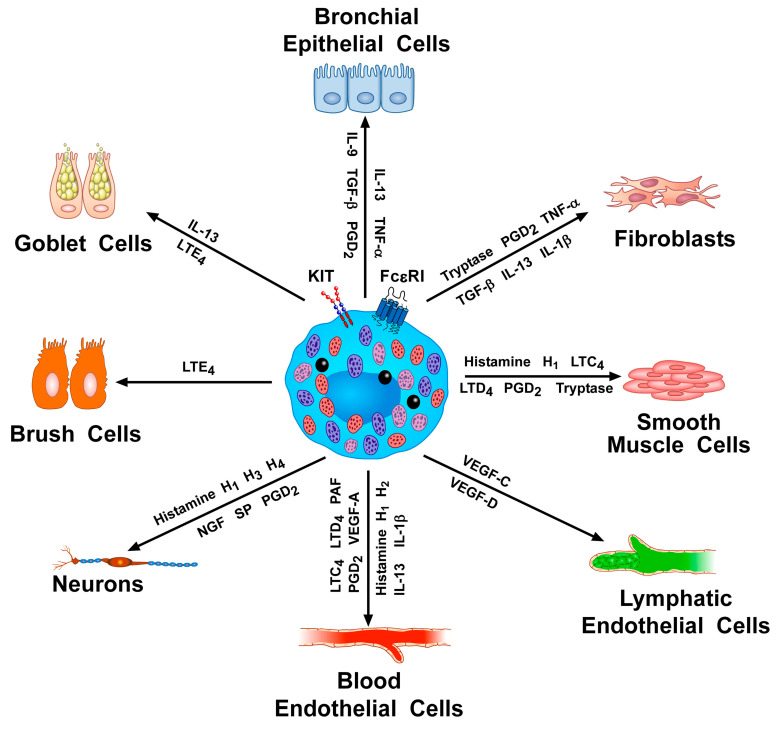
Schematic representation of the multiple interactions between human lung mast cells and various non-immune cells through the release of mediators. Mast cells can interact with bronchial epithelial cells (IL-13, TNF-α, IL-9, TGF-β, and PGD_2_), brush cells (LTE_4_), fibroblasts (tryptase, PGD_2_, TNF-α, TGF-β, IL-13, and IL-1β), smooth-muscle cells (histamine, LTC_4_, LTD_4_, PGD_2_, and tryptase), goblet cells (IL-13 and LTE_4_), blood endothelial cells (histamine, LTC_4_, LTD_4_, PGD_2_, PAF, VEGF-A, IL-13, and IL-1β), lymphatic endothelial cells (VEGF-C and VEGF-D), and neurons (histamine, NGF, SP, and PGD_2_). Modified with permission from Varricchi 2019 [151].

Figure 2 schematically illustrates the multiple interactions between HLMCs and several cells of the innate and adaptive immune system through the release of mediators. HLMCs can also interact with non-immune cells involved in bronchial asthma (Figure 3).

## 4. Mast Cell-Targeted Treatments for Bronchial Asthma

### 4.1. Histamine Receptors

Although second-generation H1 antihistamines are widely used for the treatment of allergic rhinitis and urticaria [152,153], their therapeutic role in asthma is marginal. Histamine H_4_ receptor mediates chemotaxis of HLMCs [154]. In preclinical models, H4 receptor antagonists (e.g., JNJ39758979, ZPL-3893787, and toreforant) exhibited some anti-inflammatory effects. Some have been tested in randomized control trials (RCTs) for allergic diseases with mixed results [155,156,157].

### 4.2. Tryptase

Circulating β-tryptase levels were increased in asthmatics independently of type 2 inflammation and associated with lesser omalizumab response [92]. MTPS9579A, a mAb that inhibits the activity of tryptase, is in a phase II trial in patients with moderate-to-severe asthma (NCT04092582). E104 and 31A.v11 are anti-tryptase mAbs showing promising effects in preclinical models of allergic reactions [92,158].

### 4.3. Prostaglandin D_2_

PGD_2_, the major cyclooxygenase mediator synthesized by HLMCs, activates the CRTh2 on T helper 2 cells (Th2 cells) [159]. Several CRTh2 antagonists [i.e., fevipiprant, timapiprant (OC-459), AZD1981, BI671800, and setipiprant] failed to show efficacy in asthma and allergic rhinitis patients. In particular, fevipiprant was not effective in phase III trials in asthmatics [160]. GB001, a novel CRTh2 antagonist, was well tolerated and resulted in some benefits in reducing asthma exacerbations [161].

### 4.4. Cysteinyl Leukotrienes

Leukotriene inhibitors (i.e., montelukast, zafirlukast, pranlukast, and zileuton) have been used with mixed results in allergic diseases. Montelukast, approved by the Food and Drug Administration (FDA) for the treatment of asthma and allergic rhinitis [162], is less effective compared to inhaled or intranasal glucocorticoids [163]. AZD5718, a reversible 5-lipoxygenase activating protein (FLAP) that suppresses leukotriene synthesis, is currently in Phase II trial for moderate-to-severe asthma treatment compared to montelukast (NCT05251259).

### 4.5. Mast Cell Cytokines and Their Receptors

IL-13 and IL-5 are produced by HLMCs [107]. Phase III studies demonstrated that two anti-IL-13 mAbs, lebrikizumab [164] and tralokinumab [165,166], did not reduce asthma exacerbation rates but did improve lung function in patients with severe asthma [166,167,168]. In contrast, dupilumab, a mAb which is a dual inhibitor of IL-4 and IL-13 through blockade of their shared IL-4Rα subunit, is approved for the treatment of severe uncontrolled asthma and chronic rhinosinusitis with nasal polyps [169,170,171]. The anti-IL-5 mAbs, mepolizumab [172,173] and reslizumab [174], and the anti-IL-5Rα mAb benralizumab [175] are approved as add-on therapy for the treatment of severe eosinophilic asthma [176]. These drugs markedly deplete blood eosinophils and decrease the frequency of asthma exacerbations and improve lung function in patients with severe uncontrolled asthma [174,177,178]. TNF-α is released by mouse mast cells [5], but its production by HLMCs is still controversial. Golimumab, a human mAb anti-TNF-α, showed an increase in adverse events and inconsistent efficacy in severe asthma patients [179].

A mAb anti-IL-17R, brodalumab, did not demonstrate efficacy in asthma [180]. Although mast cells are a major source of several cytokines, their production of other cytokines (i.e., IL-17A) may be selectively restricted to mast cell subtypes (e.g., synovial and skin mast cells [181]). Mast cells are also a source of IL-23 and express IL-23R [181]. Risankizumab, a mAb anti-IL-23, showed increased asthma worsening in a phase 2a trial [182].

### 4.6. Alarmins and Their Receptors

There is increasing evidence that bronchial epithelial cells represent not only a physical barrier but also an immune organ, which plays a central role in asthma pathobiology [183,184]. TSLP, IL-33, and IL-25 are upstream epithelial-derived cytokines, collectively known as alarmins [40,43,185]. These cytokines also activate downstream a broad range of cellular targets, including mast cells, to propagate the release of several cytokines involved in asthma [40].

### 4.7. Tezepelumab

**Figure 4:**
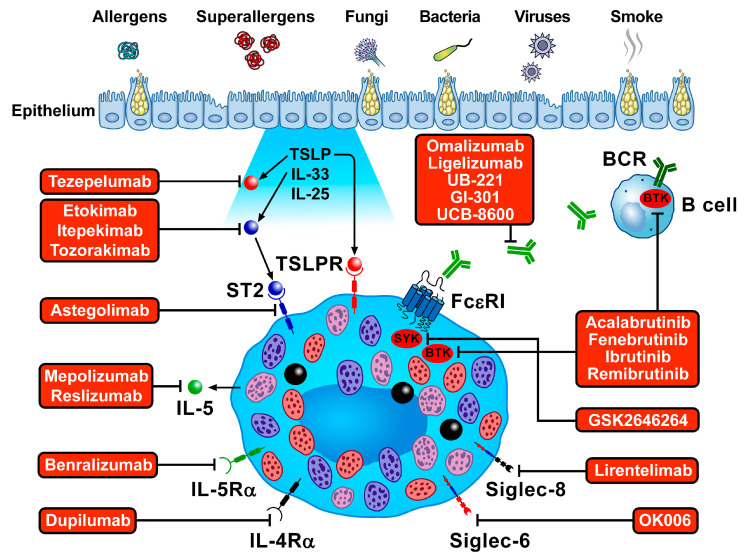
Schematic representation of mast cell-targeted biologics and drugs approved, under development, or potentially useful for asthma treatment. Several immunological stimuli (allergens, super allergens, bacterial, fungal, and viral products, smoke extracts) can damage bronchial epithelial cells to release alarmins, such as thymic stromal lymphopoietin (TSLP) [187,196], IL-33 [196,197], and IL-25 [196,198,199]. Monoclonal antibodies (mAbs) targeting TSLP (tezepelumab) [188,189], IL-33 (etokimab, itepekimab, tozorakimab) [200,201] (NCT04570657), or the IL-33 receptor ST2 (astegolimab) [202], have been approved or are under investigation for asthma treatment. mAbs targeting IL-5, produced by human mast cells [203], such as mepolizumab [204,205,206,207], reslizumab [208,209], or its receptor IL-5Rα (benralizumab) [175,210,211], have been approved for the treatment of eosinophilic asthma. Dupilumab blocks IL-4Rα and is approved for severe asthma [169,170,171]. Omalizumab was the first mAb approved for asthma treatment [212]. Novel mAbs anti-IgE (ligelizumab, UB-221, GI-301, UCB-8600) are under investigation for allergic disorder treatment [213] (NCT05298215; NTC04444466). Other strategies to treat mast cell-driven disease include inhibitors of spleen tyrosine kinase (SYK) (GSK2646264), Bruton’s tyrosine kinase (BTK) (acalabrutinib, fenebrutinib, ibrutinib, remibrutinib), and Janus kinase (JAK) inhibitors (GDC-0214) [214]. Another strategy to inhibit or deplete mast cells is the use of mAb targeting Siglec-8 (lirentelimab, also known as AK002) (NCT03379311; NCT03436797; NCT04322708) or Siglec-6 (OK006) [215].

Tezepelumab is a human IgG2λ mAb, which binds to TSLP, which is involved in different asthma phenotypes [40]. TSLP is overexpressed by the airway epithelium of asthmatics [115]. TSLP levels are increased in the BAL fluid of asthmatics [186] and serum during asthma exacerbations [187]. Tezepelumab was recently approved by the FDA and European Medicines Agency (EMA) for severe asthma treatment with no phenotype or biomarker limitations. In RCTs, tezepelumab reduced annual exacerbation rates regardless of blood eosinophil count, with an increase in prebronchodilator FEV_1_ compared to the placebo group [188,189]. In two different RCTs, tezepelumab reduced AHR, suggesting an effect on lung mast cell activation [190,191]. TSLP can promote airway remodeling in asthma through different mechanisms: it activates human lung fibroblasts [192] and causes angiogenic and lymphangiogenic factor release from HLMs [193]. In the phase II CASCADE study, the effects of tezepelumab on airway remodeling were examined in moderate-to-severe asthmatics [190]. Tezepelumab reduced airway submucosal eosinophils compared to placebo. A human mAb anti-TSLP (HBM9378) [194] and an inhaled antibody fragment against TSLP (CSJ117) [195] (NCT03138811; NCT04410523; NCT04946318) are under development for asthma treatment. Figure 4 schematically illustrates the inhibition of mast cell activation by different biologics and drugs.

### 4.8. Itepekimab

Itepekimab is a human IgG4 mAb that binds to IL-33. In a phase 2 trial, the safety and efficacy of itepekimab, dupilumab, itepekimab plus dupilumab, or placebo were compared in moderate-to-severe asthmatics [200]. Loss of asthma control was similar in the three groups but better than in the placebo. Itepekimab and dupilumab monotherapies increased pre-bronchodilator FEV_1_, reduced peripheral blood eosinophils, and improved asthma control and quality of life compared to placebo.

### 4.9. Astegolimab and Etokimab

Astegolimab is a human IgG2 mAb that targets ST2, the IL-33 receptor, and blocks IL-33 signaling [46,202]. In the phase 2b ZENYATTA study, astegolimab was well-tolerated and reduced the number of exacerbation rates in severe asthma patients [202]. Astegolimab did not significantly modify FEV_1_ compared to placebo in the entire population of asthmatics. FEV1 improvement appeared to be higher in patients with low blood eosinophils.

Etokimab (ANB020) is a humanized mAb that binds to IL-33. A preliminary study found that etokimab has the potential to desensitize subjects allergic to peanuts [201].

### 4.10. Tozorakimab

Tozorakimab (formerly MEDI3506) is a mAb that binds to IL-33 [216]. RCTs are evaluating the safety and efficacy of tozorakimab compared to placebo in adults with moderate-to-severe asthma (NCT04570657) and chronic obstructive pulmonary disease (COPD) with a history of exacerbations (NCT05166889).

mAbs blocking IL-25 have shown beneficial effects in a mouse model of allergic asthma [217].

### 4.11. FcεRI and IgE

Omalizumab, a humanized IgG1-k mAb that binds to Fcε, was the first mAb approved by the FDA for the treatment of patients with moderate and severe asthma [218]. It binds to free IgE and inhibits the IgE–FcεRI interaction by preventing the binding of IgE to FcεRI on human mast cells and basophils. Omalizumab also downregulates FcεRI expression [219]. Omalizumab did not improve FEV_1_ in RCTs [220,221,222], but there is some evidence that it can improve FEV_1_ in real-life settings [212,222]. It reduces asthma symptoms and exacerbations [223,224,225].

Ligelizumab is a second-generation humanized anti-IgE mAb, which has a higher affinity for the Cε3 domain of IgE compared to omalizumab and may affect IgE production by B cells [213]. Ligelizumab failed to meet the primary endpoints in phase II clinical trials of asthma, and it was discontinued (NCT02075008, NCT02336425). The safety and efficacy of ligelizumab are presently investigated in chronic urticaria (NCT05024058, NCT04513548, NCT03580356, NCT03580369, NCT02477332, NCT04903613). There are several promising compounds targeting FcεRI and/or IgE under investigation. GI-301, an IgE trap-Fc fusion protein, and the anti-IgE mAb UB-221 showed higher affinity to IgE compared to omalizumab (NCT05298215). Combined treatment with omalizumab and omalizumab-resistant IgE–Fc fragment (IgE-R419N-Fc3-4 mutant) caused more inhibition of basophil activation than either agent alone [226]. It has been proposed that exon skipping of the β-subunit of FcεRI in mast cells eliminated FcεRI expression and function in these cells [227].

### 4.12. Intracellular Signaling Pathways

Several promising small molecular weight compounds target intracellular signaling pathways, including spleen tyrosine kinase (SYK), Bruton’s tyrosine kinase (BTK), and Janus kinase (JAK), to block mast cell activation [214,228,229]. SYK inhibitors (i.e., fostamatinib, LAS189386, TAS05567, BAY61-3606) and aerosolized SYK antisense oligodeoxynucleotides block mast cell degranulation and inhibit models of allergic disorders [230,231,232,233]. SYK inhibitors also inhibit IgE-mediated contraction of human lung slices and histamine and leukotriene release [234]. The intranasal SYK inhibitor R112 improved symptoms in seasonal rhinitis patients [235,236].

Several BTK inhibitors are used for the management of hematological tumors [237] and are in development for the treatment of mast cell-driven diseases, including acalabrutinib for anaphylaxis [238] (NCT05038904), remibrutinib for CSU and food allergy (NCT05432388, NCT05032157, NCT05170724, NCT05513001), fenebrutinib for CSU (NCT036933625), and ibrutinib for food allergy [239] and anaphylaxis (NCT03149315). Concern has risen regarding the risk of cardiovascular adverse events associated with BTK inhibitors [237].

A phase I study assessed the safety and efficacy of GDC-0214, an inhaled JAK inhibitor, in adults with mild asthma [214]. This compound caused a dose-dependent reduction in fractional exhaled nitric oxide (FeNo) in patients with mild asthma. Additional studies on the effects of JAK inhibitors are expected for asthma treatment [240].

### 4.13. Silencing Mast Cells

Mast cells display several inhibitory receptors (i.e., Siglec-8, Siglec-6, CD200R, CD300a, and FcγRIIb) which inhibit mast cell activation [71]. An anti-Siglec-8 antibody inhibited anaphylaxis in humanized mice and IgE-dependent and IgE-independent activation of human mast cells in lung tissues [241,242,243]. Lirentelimab (AK002), a humanized anti-Siglec-8 mAb, showed promising activity in eosinophilic gastritis and duodenitis [244]. RCTs in eosinophilic esophagitis (NCT04322708), allergic conjunctivitis (NCT03379311), chronic urticaria (NCT03436797), and indolent systemic mastocytosis (NCT02808793) are ongoing. Lirentelimab reduced circulating eosinophil and tissue mast cells in eosinophilic gastrointestinal disease patients [245]. Lirentelimab depletes sputum eosinophils from asthmatic subjects and inhibits FcεRI-mediated HLMC activation [242]. Lirentelimab is presently under investigation in patients with atopic dermatitis (NCT05155085), CSU (NCT05528861), and eosinophilic duodenitis (NCT04856891).

AK006, a humanized IgG1 agonistic Siglec-6 mAb, inhibits mast cell activation in vitro. Interestingly, co-culturing human mast cells with macrophages in the presence of AK006 induces antibody-dependent phagocytosis of mast cells [215]. These findings represent a novel strategy to selectively reduce mast cells via Siglec-6 targeting.

LY3454738, a CD200R agonist, is under development for atopic dermatitis and CSU. Bispecific antibodies that cross-link either IgE [246] or KIT [247] and CD300a, and co-aggregate FcεRI with FcγRIIb, inhibited FcεRI-induced or KIT-induced signaling. An engineered protein inhibitor, designed ankyrin repeat protein (DARPin) E2-79 blocks IgE- FcεRI interactions and favors the dissociation of preformed ligand (IgE)-FcεRI complexes. Anti-IgE DARPin-Fc fusion protein inhibits allergen-induced basophil activation [248,249].

### 4.14. Depleting Mast Cells

Human mast cells express high levels of KIT throughout their development [16]. Activation of KIT by SCF influences several aspects of mast cell responses. Dysregulation of the SCF/KIT pathway markedly alters mast cell homeostasis. For instance, loss-of-function mutations in SCF or KIT result in mast cell deficiency; in contrast, gain-of-function mutations in KIT lead to mast cell hyperplasia and activation, as found in mastocytosis [250,251,252]. The blockage of the SCF/KIT pathway has been investigated in several models of allergic disorders [253,254,255,256,257]. A bispecific antibody cross-linking KIT and CD300a [247] inhibit SCF-induced human mast cell differentiation and survival and skin reactions induced by SCF in mice [247].

**Figure 5:**
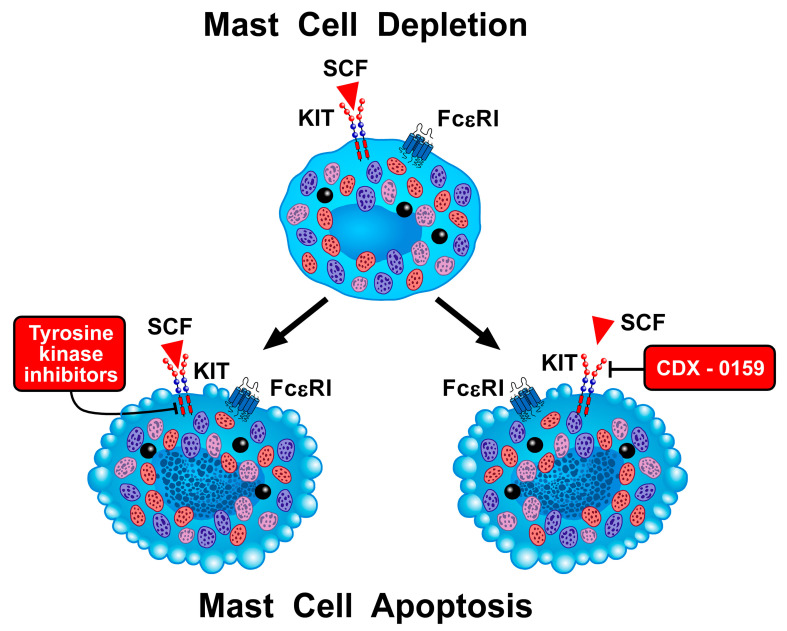
Schematic representation of two different strategies to deplete mast cells. Several classes of KIT-associated tyrosine kinase inhibitors (TKIs) are now available [16,260,261]. Preliminary evidence indicates that prolonged administration of imatinib [262] and masitinib [263,264] can influence airway hyperresponsiveness or reduce asthma exacerbations in asthma patients. These beneficial effects have been tentatively attributed to the inhibition of mast cell activation and/or depletion of mast cells. Another approach to induce mast cell apoptosis is through blockage of SCF-KIT interaction. CDX-0159 is a mAb that targets the extracellular dimerization domain of KIT [258,259] and causes a marked reduction of peripheral blood tryptase, suggesting systemic mast cell depletion (NCT04146129).

Mast cell apoptosis can be achieved via neutralization of the effects of SCF and/or blockage of its receptor KIT (CD117) (Figure 5). CDX-0159 (Celldex Therapeutics, NJ, USA) is a humanized mAb that binds to the extracellular dimerization domain of KIT [258,259]. This mAb is under investigation in CSU (NCT04538794) and chronic inducible urticaria (NCT04548869). In a phase Ia trial, CDX-0159 administration showed a favorable safety profile and caused a marked reduction of peripheral blood tryptase, suggestive of systemic mast cell depletion (NCT04146129). It remains to be evaluated whether this mAb may work in experimental models of asthma [256].

Another approach to block KIT signaling in mast cells is to use specific tyrosine kinase inhibitors (TKIs) (Figure 5). There are several classes of KIT-targeting TKIs, which display distinct pharmacologic characteristics on human mast cells in vitro [260]. KIT-targeting drugs can inhibit mast cell activation and mediator-induced symptoms in allergic disorders [265,266,267,268]. There are very preliminary data on the in vivo efficacy of KIT-specific or multitargeted TKIs in the treatment of patients with severe allergic disorders (e.g., severe asthma). The administration of masitinib to patients with severe glucocorticoid-dependent asthma was associated with steroid-sparing effects [264]. Imatinib did not influence lung function. In another study, imatinib reduced airway hyperresponsiveness in patients with severe asthma compared to controls [262].

In a phase III trial, masitinib reduced asthma exacerbations compared to placebo in severe asthma patients [263]. Avapritinib (BLU-285), a potent inhibitor of mutant KIT and PDGFRA with activation loop mutations, induces mast cell cytoreduction and remission in the majority of advanced systemic mastocytosis patients [269,270]. It should be emphasized that some of these TKIs also inhibit IgE-dependent basophil activation [271,272,273]. This is relevant because basophils play a role in allergic disorders [88,274]. Future studies should evaluate the safety and efficacy of imatinib, masitinib, and possibly newer TKIs in patients with different phenotypes of severe asthma.

## 5. Discussion and Conclusions

Human mast cells were identified and named over 140 years ago by Paul Ehrlich [1]. IgE was discovered by Kimishige and Teruko Ishizaka [275] and Gunnar Johansson [276]. The approval of omalizumab, the first mAb anti-IgE for the treatment of asthma in 2003, was a breakthrough in the treatment of patients with mast cell-driven diseases, such as asthma and CSU. Since then, several biologics targeting mast cells directly or indirectly have been approved for the treatment of severe asthma. In particular, mAbs targeting IL-5 (mepolizumab [204,205,206,207] and reslizumab [208,209]), IL-5Rα (benralizumab) [175,210,211], IL-4Rα (dupilumab) [169,170,171,212], and TSLP (tezepelumab) [188,189] have been demonstrated to reduce annual exacerbation rates and also certain features (e.g., FEV_1_) of airway remodeling in severe asthmatic patients. The efficacy and safety of the above mAbs have been recently discussed in detail [142]. Collectively, these clinical findings support the involvement of lung mast cells in central features of severe asthma.

Several promising mast cell-targeted biologics, such as mAbs anti-IL-33 [200] (NTC04570657), anti-ST2 [202], anti-Siglec-8 (NCT03379311; NCT03436797; NCT04322708), and CDX-0159 (NCT04146129) have entered clinical development in asthma or allergic disorders. Moreover, several classes of drugs silencing or depleting mast cells (e.g., TKIs) have shown promising results in patients with severe uncontrolled asthma [262,263,264].

We are going through an exciting and promising era for understanding human mast cell biology. However, we must consider that many aspects of mast cell biology and their complex phenotypic and functional heterogeneity remain largely unknown. Mast cells are exposed to their local environment that, over time, can modify their phenotype and biochemical machinery [277]. More studies using novel techniques (e.g., single-cell mRNA seq, CyTOF) will more accurately reveal mast cell heterogeneity [278,279]. These techniques will contribute to identifying the role of mast cell subtypes in different asthma phenotypes. Another level of complexity derives from the species differences in extrapolating findings from mouse mast cell models to human settings [19,43].

Human mast cells and basophils have some similarities (e.g., FcεRI) but also striking differences [43]. Basophils have been recently identified in the human lung [26,27,280], where they play a prominent role in macrophage differentiation [281]. Macrophages represent the most prominent immune cells in human lung tissue [282,283]. There is also evidence that basophils and their mediators (i.e., IL-4, IL-13) play a role in Th2 and M2 polarization in allergic asthma [43]. Likely, some biologics that primarily target mast cells (e.g., omalizumab, mepolizumab, benralizumab) may also target human basophils [284,285].

Mast cells and their mediators play homeostatic and protective roles in several pathophysiological conditions [19,286]. Moreover, several normal cell types, such as germ cells, hematopoietic stem cells, and melanoblasts, express KIT, and the chronic administration of TKIs and mAbs targeting KIT may be associated with long-term adverse effects. Caution will be necessary in the future when drugs able to markedly reduce tissue mast cells in humans will be available for the treatment of mast cell-driven diseases.

## Figures and Tables

**Table 1:** Preformed and Newly Synthesized Mediators Released by Human Lung Mast Cells.

Mediator	Properties	References
Preformed		
Histamine	Preformed in cytoplasmic granules of human mast cells (≅3 pg/cell) and basophils (≅1 pg/cell).	[64,65]
β-tryptase	A tetrameric serine protease, abundant in secretory granules of human mast cells. Tryptase^+^ mast cells are increased in asthmatics bronchial tissue, and their numbers correlate with airway hyperresponsiveness. Tryptase concentrations in bronchoalveolar lavage (BAL) fluid correlates with asthma severity.	[89,90,91,92]
Chymase	A chymotrypsin-like serine protease stored in human mast cell secretory granules causes matrix destruction and inflammation.	[93,94]
Cathepsin G	A serine protease that controls the functional state of immune cells.	[93]
Carboxypeptidase A3	It cleaves several proteins.	[95]
Granzyme B	A protease involved in the induction of target cell death.	[96]
Matrix metalloproteinases (MMPs)	A family of extracellular proteinases.	[97]
**Lipid Mediators**		
Cysteinyl leukotriene C_4_ (LTC_4_)	Human lung mast cells (HLMCs) synthesize LTC_4,_ a potent bronchoconstrictor acting through the activation of cysteinyl leukotriene receptor 1 (CysLTR1) and CysLTR2.	[98,99]
Prostaglandin D_2_ (PGD_2_)	It activates the CRTh2 receptor on several immune cells.	[100,101]
Platelet-activating factor (PAF)	A phospholipid with proinflammatory and vasoactive effects.	[102]
**Cytokines**		
Stem cell factor (SCF)	They exert several proinflammatory and immunomodulatory effects.	[37]
TNF-α		[103]
IL-1β		[104]
IL-3		[105,106]
IL-5		[107]
IL-6		[108]
IL-9		[109]
IL-10		[57]
IL-11		[108]
IL-13		[109,110]
IL-16		[111]
IL-22		[112]
Thymic stromal lymphopoietin (TSLP)		[113,114,115]
IL-25/IL-17E		[116]
Granulocyte-macrophage colony-stimulating factor (GM-CSF)		[107]
Vascular endothelial growth factor (VEGF)		[117]
Fibroblast growth factor 2 (FGF-2)		[118]
Nerve growth factor (NGF)		[119]
Amphiregulin		[120,121]
**Chemokines**		
CXCL8/IL-8, CCL1/I-309, CCL2/MCP-1, CCL3/MIP-1α, CXCL1/GRO-α, CXCL10/IP-10	They exert several chemotactic and proinflammatory effects.	[122,123,124]
**Angiogenic factors**		
VEGF-A	The main angiogenic factor released by HLMCs. VEGFs released by macrophages, basophils, and neutrophils contribute to mast cell infiltration in bronchial asthma.	[56,117,125,126,127,128]
Angiopoietins (ANGPT1 and ANGPT2)	ANGPTs are involved in blood vessel formation and are released by HLMCs.	[129,130,131,132]
LTC_4,_ LTD_4_	Non-canonical angiogenic factors.	[133]
**Lymphangiogenic factors**		
VEGF-C, VEGF-D	The main lymphangiogenic factor released by HLMCs.	[58]

## Abbreviations

AHR: airway hyperresponsiveness; ANGPT, angiopoietin; BAL, bronchoalveolar lavage; BTK, Bruton’s tyrosine kinase; C5aR, C5a receptor; CADM1, cell adhesion molecule 1; CRH, corticotropin-release hormone; CSU, chronic spontaneous urticaria; CysLTR1, cysteinyl leukotriene receptor 1; CyTOF, Cytometry by time-of-flight; DARPin, designed ankyrin repeat protein; DC, dendritic cell; FDA, Food and Drug Administration; EMA, European Medicines Agency; FeNO, fractional exhaled nitric oxide; FLAP, 5-Lipoxygenase activating protein; HLMC, human lung mast cell; HSMC, human skin mast cell; JAK, Janus kinase; LTC_4_, cysteinyl leukotriene C_4;_ mAb, monoclonal antibody; HMC, human mast cell; MC_T_, tryptase^+^ mast cell; MC_TC_, tryptase^+^, chymase^+^ mast cell; MMP, matrix metalloprotease; MRGPRX2, Mas-related G protein-coupled receptor X2; PAF, platelet-activating factor; PAR2, protease activated receptor 2; PGD_2_, prostaglandin D_2_; RCT, randomized control trial; SCF, stem cell factor; SYK, spleen tyrosine kinase; TKI, tyrosine kinase inhibitors; TLR, toll-like receptor; TSLP, thymic stromal lymphopoietin; TSLPR, thymic stromal lymphopoietin receptor; VEGF, vascular endothelial growth factor; VEGFR, vascular endothelial growth factor receptor.
